# Tobacco and Nicotine Products Adverse Health Events: Findings From the FDA Safety Reporting Portal

**DOI:** 10.1177/1179173X241279674

**Published:** 2024-08-28

**Authors:** Steven Alec Barrientos, Danny Dabroy, Mohammad Ebrahimi Kalan, Linnea Irina Laestadius, Ziyad Ben Taleb

**Affiliations:** 1 Department of Kinesiology, College of Nursing and Health Innovation, 12329 The University of Texas at Arlington, Arlington, TX, USA; 2 School of Health Professions, 6040 Eastern Virginia Medical School, Norfolk, VA, USA; 3 Joseph J. Zilber College of Public Health, 14751 University of Wisconsin-Milwaukee, Milwaukee, WI, USA

**Keywords:** tobacco products, adverse health events, nicotine products, food and drug administration safety reporting portal, food and drug administration tobacco product problem reports, e-cigarettes, electronic nicotine delivery systems, adverse effects, United States food and drug administration, health surveillance

## Abstract

**Introduction:**

The FDA oversees regulatory aspects of all U.S. tobacco products. Understanding the impact of emerging health trends and incidents associated with various tobacco and nicotine products is vital for public health. This study utilizes the FDA’s Tobacco Product Problem Reports (TPPRs) to characterize and track adverse health events (AHEs) associated with tobacco and nicotine products over time, considering the impact of EVALI and the COVID-19 pandemic.

**Methods:**

FDA TPPRs from 2017-2022 provided information on AHEs related to various tobacco products. After data cleaning, 839 reports were categorized by two independent coders based on affected health category, frequency of AHEs reports, and proportion of AHEs per each health category. Additionally, variations in AHEs over time were assessed, considering major health events like EVALI and the COVID-19 pandemic.

**Results:**

Among the 839 reports, electronic cigarettes (e-cigarettes) were the primary product of concern, comprising 90.6% (n = 760) of all reports, surpassing traditional cigarettes (5.1%; n = 43) and other products. Notably, 45.6% of reports (n = 383) identified the neurological system as the most frequently mentioned health category, each reporting at least one AHE. This was followed by the respiratory (39.1%; n = 328) and digestive (10.7%; n = 90) systems. Among all reported AHEs, respiratory system issues were most frequent (25.9%; n = 512), closely followed by neurological (25.2%; n = 499) and digestive (6.6%; n = 131) concerns. Most reports occurred in 2019 (65.7%; n = 551), coinciding with the EVALI outbreak, with a subsequent decline post-Q3 2019, highlighting the potential impact of specific health crises on reporting trends.

**Conclusion:**

E-cigarettes dominated adverse health reports, particularly affecting the neurological and respiratory systems, with a peak in 2019. Our findings provide insights to regulatory entities and future research, enhancing understanding of AHEs in lesser-explored bodily systems, such as the neurological and digestive systems. This study emphasizes the need for ongoing and improved surveillance of emerging tobacco products to protect public health.

## Introduction

Following the enactment of the Family Smoking Prevention and Tobacco Control Act in 2009, the U.S. Food and Drug Administration (FDA), operating through its Center for Tobacco Products, was given regulatory authority over the production, sales, distribution, and advertising of all tobacco products in the United States.^1^

As part of their comprehensive approach to monitoring tobacco and nicotine products, the FDA has established a dedicated reporting system to track adverse health events associated with these products. Central to this comprehensive reporting system is the FDA Safety Reporting Portal (SRP), designed to monitor various tobacco products, including electronic cigarettes, traditional cigarettes, pipe tobacco, heated tobacco products, roll-your-own cigarettes, dissolvable products, cigars, chewing tobacco, and more. The FDA SRP serves as a self-reporting platform accessible to product users, health professionals and manufacturers.^2^ Its main objective is to collect information regarding any adverse health or quality issues believed to be linked to a specific tobacco product.

These data are then compiled into the Tobacco Product Problem Reports (TPPRs), which are quarterly summaries documenting adverse health effects associated with tobacco and nicotine products.^2^ The TPPRs are readily accessible to researchers and the public, simplifying access to commonly reported potential issues with tobacco products. Moreover, this proactive approach aids the FDA in better understanding and addressing potential risks associated with these products. Furthermore, the FDA TPPRs serve as the basis for in-depth research into emerging health trends and events related to various tobacco and nicotine products. These data are critical for assessing manufacturer compliance with regulations, identifying irregularities, and tracking adverse health trends associated with nicotine and tobacco products, thereby ensuring public safety.

The importance of these data becomes clear when seeing specific examples of new and emerging tobacco products, such as electronic cigarettes (e-cigarettes), which lack extensive longitudinal research on safety and health effects. Many users hold a misguided perception of harmlessness when using e-cigarettes compared to traditional combustible cigarettes or other tobacco products.^3^ However, the use of e-cigarettes has been linked to a spectrum of adverse health events, including but not limited to conditions such as popcorn lung, EVALI (e-cigarette or vaping product use-associated lung injury), incidents of device explosion, nicotine poisoning, and various other health concerns.^4–7^ This highlights the importance of understanding the adverse health events associated with these products, particularly considering that 2.13 million middle and high school students report current e-cigarette use.^8^

Moreover, it is imperative to thoroughly investigate adverse health events linked with both traditional and other emerging tobacco products in the United States. This emphasis extends to a focus on the younger demographic, as they often exhibit a tendency to experiment with various forms of tobacco.^9^ Understanding and addressing the potential risks and adverse health events associated with these products among young individuals is paramount for developing effective public health interventions and preventive measures. Furthermore, it is essential to examine the potential health impacts of emerging tobacco products, such as hookah and cigars/cigarillos, especially in the context of their heavy promotion on social media platforms.^10^

In this study, we aim to evaluate the FDA’s TPPRs to understand and track adverse health events related to tobacco and nicotine products from 2017 through 2022. This information will improve our understanding of how these products impact health and can guide future public health efforts and regulatory actions.

## Methods

The research team extracted data from the FDA’s 2017-2022 TPPRs, sourced from the FDA SRP. These TPPRs contain extensive information, including report identification numbers (ID), submission dates, details of health events, types of products used and descriptions of product problems.^2^

### Data collection

Initiating on March 15, 2023, the data extraction process involved downloading the TPPRs from the FDA SRP, resulting in a total of 1138 reports. Subsequently, researchers conducted data cleaning procedures. As our study exclusively utilized publicly available and unidentified data, it was deemed exempt from IRB review.

**Figure 1.**
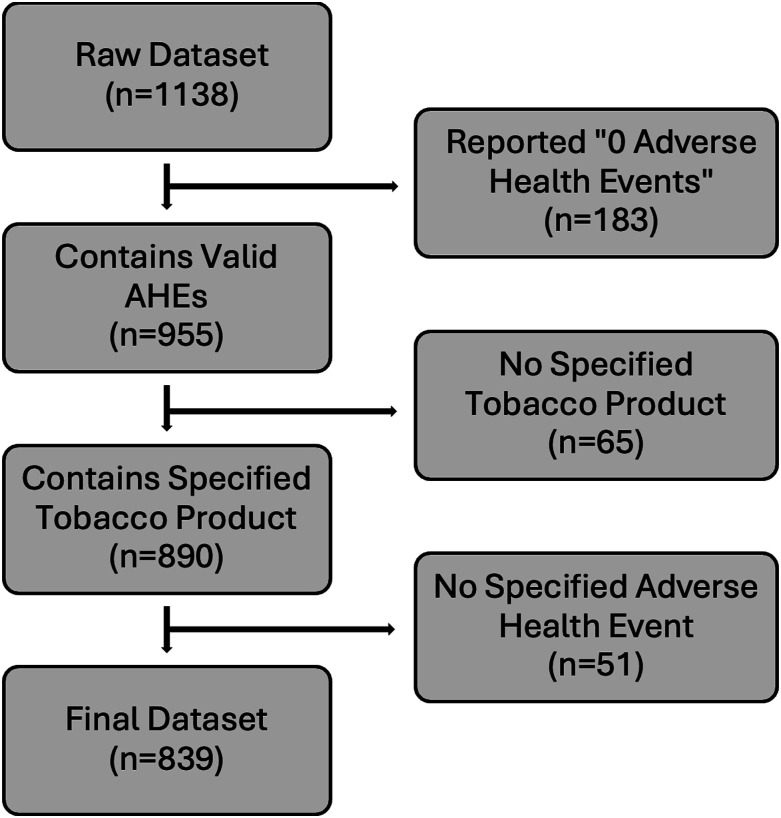
Data cleaning process flowchart.

Following the data extraction process, reports with missing, inadequate, or insufficient information that could hinder the description of associations between products and adverse health events were systematically excluded, as depicted in Figure 1. Following the elimination of these specified reports, the final sample consisted of 839 reports, revealing a total of 1977 adverse health events.

### Health category selection

Reports were categorized according to various criteria, including the date of submission, report ID, number of reported adverse health events, and the specific tobacco product mentioned. Additionally, each report was categorized based on which health categories were affected by the tobacco product or associated event. For example, the “respiratory” category could involve symptoms such as “coughing” or “shortness of breath”, indicative of potential respiratory system issues. Similarly, an adverse health event categorized under “psychological” could include symptoms like “mood swings” or “anxiety attacks”, suggesting disturbances in mental health. These categories, along with others such as musculoskeletal, digestive, physical trauma, integumentary, oral/dental, cardiovascular, neurological, death, laboratory test/treatment, and addiction, were precisely defined with specific criteria, as outlined in Appendix 1.

It is important to note that when categorizing each adverse health event, the categories were mutually exclusive, meaning each event was assigned to only one designated health category. However, each adverse health report was not mutually exclusive and could be defined by multiple health categories based on the adverse health events present within each report. Events that did not meet any categorical criteria were labeled as “nonspecific”, as detailed in Appendix 2.

### Coding process and data analysis

To categorize the adverse health events into distinct health categories, a pilot sample of 200 reports was coded. During this phase, two independent coders met regularly to discuss emerging health categories and to agree on the final selection of these categories. Following this initial phase, a thorough re-coding was undertaken to ensure the accurate assignment of codes to these reports. Afterward, the remaining 639 reports were evenly distributed and randomly assigned to two researchers for independent coding. In instances of coding disagreement, a third independent rater was consulted to reevaluate the report. A meeting was then convened involving all three raters to discuss discrepancies and reach consensus.

To ensure interrater reliability, a 10% (n = 88) random sample of reports was double coded by two researchers. This process aimed to measure the level of category agreement between the coders, ensuring that both assigned the same code to each variable. By enhancing consistency and minimizing potential biases, this approach bolstered the credibility of the coding process.^11,12^ Statistical analysis showed an average interrater reliability score (Cohen’s weighted Kappa) of 0.9, with values ranging from 0.794 to 1.000 (*P* < 0.01), indicating a notably high level of agreement between the two raters, as depicted in Appendix 3.

Descriptive statistics of the study sample were summarized with means or proportions. All analyses were conducted using IBM SPSS V.29 and Microsoft Excel.

## Results

Table 1 presents the distribution of reports and adverse health events associated with different types of tobacco and nicotine products. Of the 839 reports, the most frequently reported product type was e-cigarettes (n = 760; 90.6%), followed by cigarettes/roll-your-own (n = 49; 5.8%), heated tobacco (n = 7, 0.8%), dissolvable/chewing tobacco (n = 7, 0.8%), pipe (n = 5, 0.6%), cigars (n = 4, 0.5%), snuff (n = 4, 0.5%), and other (n = 3, 0.4%). Of the 1977 adverse health events, the majority were attributed to e-cigarettes (n = 1846; 93.4%), followed by cigarettes/roll-your-own (n = 76; 3.8%), heated tobacco (n = 15; 0.8%), cigars (n = 13, 0.7%), dissolvable/chewing tobacco (n = 12, 0.6%), pipe (n = 6, 0.3%), snuff (n = 6, 0.3%), and other (n = 3; 0.2%).

**Table 1.** Tobacco Product Type by Total Reports and Adverse Health Events Reported.

Product Type	Total Reports	Percent	Adverse Health Events	Percent
E-cigarette	760	90.6	1846	93.4
Cigarette/Roll-your-own	49	5.8	76	3.8
Heated tobacco^a^	7	0.8	15	0.8
Dissolvable/chewing tobacco^b^	7	0.8	12	0.6
Pipe	5	0.6	6	0.3
Cigar^c^	4	0.5	13	0.7
Snuff	4	0.5	6	0.3
Other^d^	3	0.4	3	0.2
**Total**	**839**		**1977**	

^a^Heat-not-burn, or dry/moist tobacco in heated tobacco system (eg, hookah/waterpipe).

^b^Loose leaf chew, plug, twist/roll, strips, sticks, orbs or gutka.

^c^Large, premium, small, or little cigar or cigarillo.

^d^Lotions, gels, or poly-use products.

**Figure 2.**
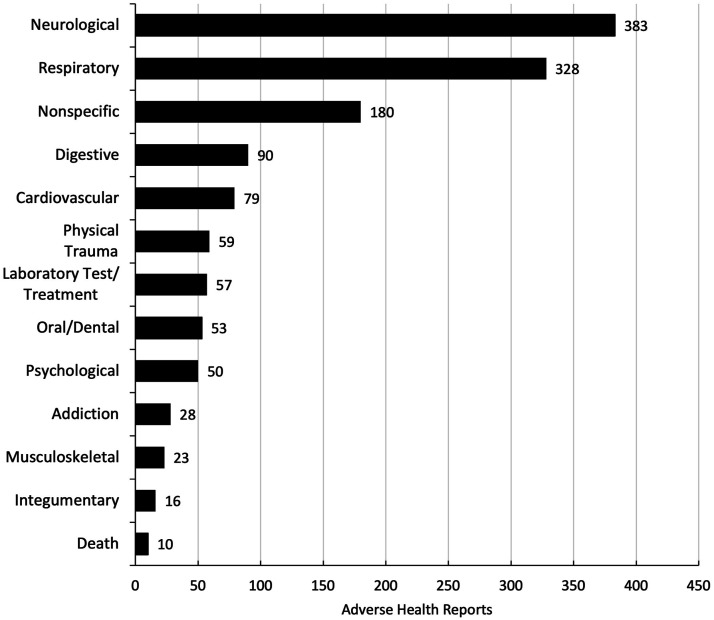
Number of adverse health reports across health categories.

A distribution of the frequency of adverse health reports by health category is shown in Figure 2. Among the 839 reports, the nervous system emerged as the most commonly affected health category (n = 383, 45.6%), followed by respiratory (n = 328, 39.1%), nonspecific (n = 180, 21.5%), digestive (n = 90, 10.7%), cardiovascular (n = 79, 9.4%), physical trauma (n = 59, 7%), laboratory test/treatment (n = 57, 6.8%), oral/dental (n = 53, 6.3%), psychological (n = 50, 6%), addiction (n = 28, 3.3%), musculoskeletal (n = 23, 2.7%), integumentary (n = 16, 1.9%), and death (n = 10, 1.2%).

**Figure 3.**
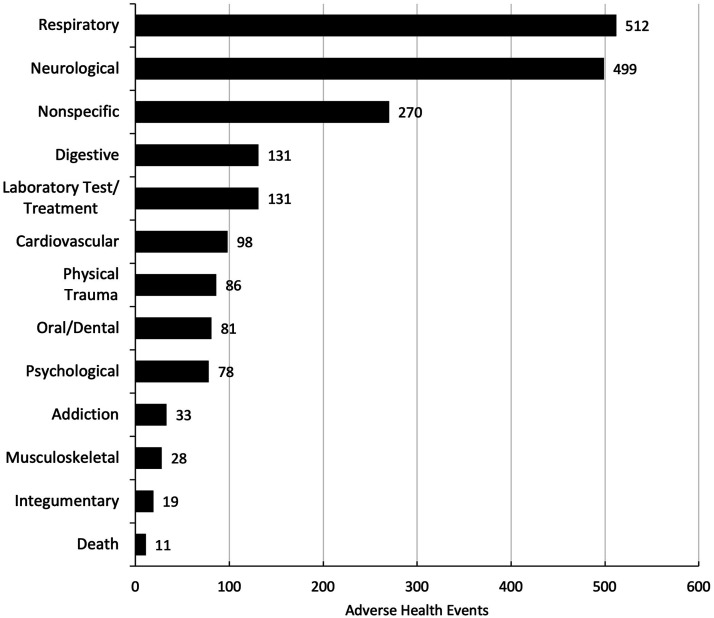
Number of adverse health events within all reports across health categories.

Across the total number of adverse health events reported (n = 1977), the respiratory system was the most frequently affected health category (n = 512, 25.9%). Following this, the nervous system was also prominently affected (n = 499, 25.2%), followed by nonspecific (n = 270, 13.7%), digestive (n = 131, 6.6%), laboratory test/treatment (n = 131, 6.6%), cardiovascular (n = 98, 5%), physical trauma (n = 86, 4.4%), oral/dental (n = 81, 4.1%), psychological (n = 78, 3.9%), addiction (n = 33, 1.7%), musculoskeletal (n = 28, 1.4%), integumentary (n = 19, 1%), and death (n = 11, 0.6%), as indicated in Figure 3.

**Figure 4.**
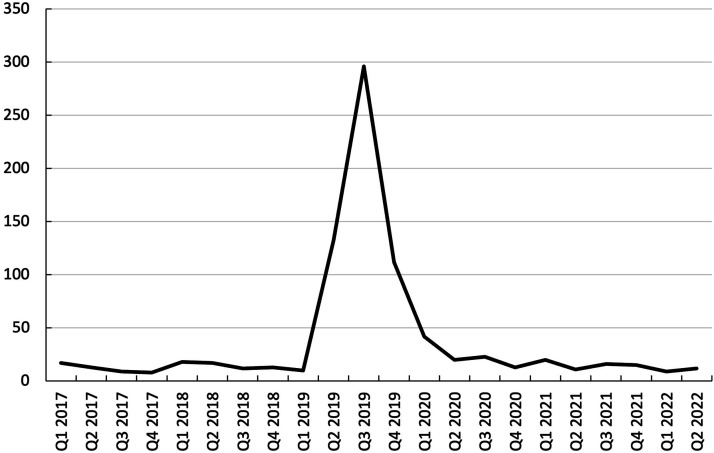
Adverse health reports by year-quarter.

Appendix 4 presents the distribution of health reports by year. The study evaluated adverse health events associated with tobacco product usage spanning from 2017 through the first half of 2022. The majority of adverse health reports were submitted in 2019 (n = 551, 65.7%), followed by 2020 (n = 98, 11.7%), 2021 (n = 62, 7.4%), 2018 (n = 60, 7.2%), 2017 (n = 47, 5.6%), and finally 2022 (n = 21, 2.5%). Specifically, quarter 3 of 2019 recorded the highest number of reports (n = 296, 35.3%), as depicted in Figure 4.

## Discussion

This study is amongst the first to delve into the characterization and tracking of adverse health events related to tobacco and nicotine products over six years, utilizing data sourced from the U.S. FDA’s TPPRs. Our findings revealed that the number of reported adverse health events associated with e-cigarettes significantly outweighed those associated with cigarettes and all other tobacco products. This observation may be influenced by the concurrent decline in cigarette usage in the U.S. and a peak in e-cigarette use in 2019, followed by a subsequent return to baseline levels.^13-15^

Interestingly, the sharp increase of adverse health events in 2019 closely aligns with the 2019 outbreak of EVALI. Though the exact cause was initially uncertain, recent investigations have largely indicated vitamin E acetate, a thickening agent present in some illicit THC-containing vaping products, as a significant contributor to EVALI cases.^13,14^ Polysubstance use (ie, nicotine and THC vaping), initial uncertainty about the causes of EVALI, and heightened media coverage and public awareness surrounding EVALI, likely contributed to the uptick in reporting of adverse health events related to e-cigarettes overall.^15,16^ Our findings underscore the need for ongoing attention in monitoring the adverse health impacts of tobacco and nicotine products, particularly in light of shifting usage patterns and emerging trends, such as the vaping of cannabis products using e-cigarette devices.^17,18^ Moreover, to accurately monitor nicotine and tobacco use, surveillance techniques must evolve to account for the diverse range of devices, substances and practices, recognizing that the term “vaping” is overly broad.

Additionally, the onset of the COVID-19 pandemic in early 2020 may have influenced the reporting of several adverse health events in the FDA TPPRs, particularly those classified as “respiratory” and “digestive”. Symptoms such as coughing, shortness of breath, and chest pain are common to both COVID-19 and adverse reactions from e-cigarette use, complicating the accurate attribution of these events, and potentially leading to the misattribution of symptoms to either COVID-19 or e-cigarette use.

Furthermore, our research found that the majority of adverse health reports associated with e-cigarettes were neurological in nature. This extends previous FDA’s findings between 2010 and 2019, during which they reported only 127 cases related to seizures and neurological problems associated with vaping.^19,20^ Vaping may deliver substances more rapidly than smoking, possibly causing increased brain irritation. Moreover, illicit substance purchases pose risks due to unknown contaminants. In a study analyzing e-cigarette liquid samples from daily users, researchers found lead, chromium, nickel, manganese, and arsenic.^21^ These neurotoxicants can cause health issues such as headaches, seizures, and other neurological complications, depending on the dosage and individual susceptibility.^22^

In our study, the high number of reports related to neurological symptoms was closely followed by frequent reporting of respiratory symptoms. The impact of e-cigarette use on the respiratory system has been documented in previous studies,^5,6,23^ and recent systematic reviews have confirmed the prevalence of adverse respiratory symptoms among exclusive e-cigarette users, dual users, and those transitioning from combustible cigarettes to e-cigarettes.^24^ The compounded problem of neurological and respiratory effects raises significant public health concerns, particularly among youth.^25^ In light of these findings, it is imperative for regulatory authorities and healthcare professionals to monitor, investigate, and accurately report not only the established respiratory effects but also the emerging neurological effects associated with e-cigarette use. This comprehensive approach is essential for strengthening public health protection, particularly among vulnerable populations such as youth.

Another interesting finding from this study, is the emerging reports of symptoms related to the digestive system among e-cigarette users. This has important implications as this aspect remains largely unexplored, with the few available studies conducted primarily in animals.^26^ It is crucial for future research to look more into how vaping affects the digestive tract, to fill in these gaps in our understanding.

This study is not without limitations. First, the adverse health events reported lacked medical confirmation, potentially leading to misreported symptoms and their subsequent misclassification into inappropriate health categories. The inclusion of self-reported submissions could introduce self-report bias, resulting in incomplete data, underreporting of multiple substance use, and over-reporting of symptoms. Additionally, our study’s reliance on voluntary submissions limits its ability to capture the full spectrum of adverse health events associated with tobacco products, potentially resulting in underreporting.^27^ Moreover, it is essential to underscore that data from the TPPRs cannot establish causality or accurately estimate incidence rates or risks.^2^ Another limitation is that the “nonspecific” category was the third most common, highlighting the difficulty in categorizing adverse health events that do not fit neatly into specific health categories. This approach was chosen to prevent inaccurate classification and maintain data integrity.

Furthermore, younger individuals may inaccurately report adverse health events due to lack of knowledge or awareness, often seeking information or advice from informal online sources such as Reddit.^28^ Additionally, since e-cigarettes can be used with both nicotine and cannabis products, some adverse health events may be associated with cannabis rather than nicotine vaping, potentially impacting findings.^29^

While the absolute number of adverse health events reported is relatively low compared to other health complications such as those associated with medications, the importance of continuous monitoring and adaptation to new and emerging nicotine and tobacco products cannot be overstated. This proactive approach to surveillance is crucial to rapid detection and lessening of events similar to the EVALI outbreak in the future. To enhance data accuracy and specificity, the FDA may consider separate reporting mechanisms for nicotine and cannabis-related adverse health effects associated with e-cigarette use. This approach would enable more precise tracking and analysis of the distinct health impacts associated with each substance, leading to better-informed regulatory decisions and public health interventions tailored to the unique risks posed by exclusive and dual consumption of nicotine and cannabis.

## Conclusion

This study contributes significantly to the literature by comprehensively characterizing and tracking adverse health events associated with tobacco and nicotine products over time. Our findings offer useful information for regulators and researchers, enhancing our understanding of how these products affect less-studied body systems, such as the neurological and digestive systems. Additionally, these findings have important clinical implications. Physicians should be watchful in asking about symptoms related to tobacco and e-cigarette use, obtaining thorough medical histories and reporting adverse health events. This underscores the need for continuous, detailed surveillance of emerging tobacco products and substances to protect public health effectively.

## Supplemental Material

Supplemental Material - Tobacco and Nicotine Products Adverse Health Events: Findings From the FDA Safety Reporting Portal Supplemental Material for Tobacco and Nicotine Products Adverse Health Events: Findings From the FDA Safety Reporting Portal by Steven Alec Barrientos, Danny Dabroy, Mohammad Ebrahimi Kalan, Linnea Irina Laestadius and Ziyad Ben Taleb in Tobacco Use Insights.
