# Supplementary material for: Tobacco and Nicotine Products Adverse Health Events: Findings From the FDA Safety Reporting Portal
Source: Tob Use Insights. 2024 Aug 28;17:1179173X241279674. doi: 10.1177/1179173X241279674 (PMC11363042; doi:10.1177/1179173X241279674)
Supplement: Supplemental Material - Tobacco and Nicotine Products Adverse Health Events: Findings From the FDA Safety Reporting Portal [file sj-pdf-1-tui-10.1177_1179173X241279674.pdf]

SUPPLEMENTARY MATERIAL

Appendix 1. Health category definitions for categorizing adverse health events.

Definitions of each health category:

1. **Respiratory**: Symptoms of, relating to or affecting gas exchange and respiration and/or the organs and tissues of the respiratory system, including parts of the upper (e.g., nose, nasal cavities, and sinuses) and lower respiratory tract (e.g., trachea, bronchi, lungs, bronchioles, and alveoli).
2. **Psychological**: Symptoms of, pertaining to or causing significant disturbances in behavior, emotion, and cognition, including mood, anxiety, personality, psychotic, eating, trauma-related, and substance abuse symptoms and disorders.
3. **Neurological**: Symptoms of, relating to or affecting sensory input, integration of data and motor output and/or the nerves and cells of the nervous system, including the central (e.g., brain and spinal cord) and peripheral nervous system (e.g., autonomic [e.g., sympathetic, and parasympathetic system] and somatic nervous system).
4. **Cardiovascular**: Symptoms of, relating to or affecting the circulation of blood pumped by the heart through the closed circuit of vessels, and/or the organs and tissues of the cardiovascular system, including the heart, blood vessels (e.g., arteries, veins, and capillaries) and blood (e.g., cells and plasma).
5. **Oral/Dental**: Symptoms of, relating to or involving the mouth, teeth, gums, tongue, throat (e.g., pharynx and larynx) or other oral-facial structures.
6. **Integumentary**: Symptoms of, relating to or affecting the bodily protection or maintenance functions of the skin and/or the organs and tissues of the integumentary system, including the skin (e.g., epidermis, dermis, and subcutaneous layer), and its associated structures (e.g., hair and nails).
7. **Physical Trauma**: Symptoms resulting from body wounds or serious injuries produced through sudden physical forces or objects from impact, violence, or accident.
8. **Digestive**: Symptoms of, relating to or affecting digestion, absorption of nutrients, and elimination of solid food waste and/or the organs and tissues of the digestive system, including parts of the gastrointestinal tract (e.g., esophagus, stomach, small intestine, large intestine, rectum, and anus) and the accessory organs of digestion (e.g., pancreas, liver, and gallbladder).
9. **Musculoskeletal**: Symptoms of, relating to or affecting movement, stability, shape, and support of the body and/or the tissues of the musculoskeletal system, including bones, joints, cartilage, ligaments, tendons, and connective tissues.
10. **Death**: Individual who has sustained either (1) irreversible cessation of circulatory and respiratory functions, or (2) irreversible cessation of all functions of the entire brain, including the brain stem.
11. **Lab test/ Treatments**: Individual who (1) undergoes clinical/laboratory confirmation testing or (2) treatments by health professionals or hospital staff.
12. **Nonspecific**: Adverse health events that are nonspecific and/or do not classify into the above-mentioned categories.

Appendix 2. Breakdown of the ‘nonspecific’ adverse health events category.

Adverse health events categorized as 'nonspecific', further delineated by secondary health categories:

1. **General Health**: Abdominal flaccidity, Abnormal loss of weight, Anemia, Appetite lost, Bleeding, Body temperature normal, Burning sensation, Burning sensation mucosal, Cancer, Chest heaviness, Chest pain (non-cardiac), Chest tightness, Chills, Chronic fever, Clostridium difficile infection, Cold, Condition worsened, Congenital immunodeficiency, Cyanosis, Cyanotic, Decreased appetite, Dehydration, Diaphoresis, Drenching sweats, Drowsiness, Edema, Energy decreased, Energy increased, Excess sweating, Fatigue, Fatigue extreme, Feeling abnormal, Feeling bad, Feeling sick, Feeling unwell, Fever, Fever of unknown origin, Flu like symptoms, Flu-like symptoms, General discomfort, General malaise, Generalised chest pain, Heavy sweating, ill-defined disorder NOS, illness, Immune system disorder, Implant infection, Incontinent, Infection, Intractable pain, Kidney failure, Lethargy, Malnutrition, Night sweats, Pain, Pain mucosal, Phlegm discolored, Seasonal allergy, Sepsis, Sepsis NOS, Septic shock, Sickness, Sputum discolored, Sweating, Sweating abnormal, Swollen lymph nodes, Transaminitis, Tiredness, Unintentional weight loss, Weakness, Weight loss.
2. **Accidents/Injuries**: Drowning, Fall, Motor vehicle accident.
3. **Auto Immune**: Autoimmune disorder, Autoimmune disorder NOS, Hashimoto's thyroiditis, Lupus erythematosus.
4. **Ear, Nose, Throat**: Ear feels clogged, Ear infection, Ear popping sensation, Hard of hearing, Hearing impaired, Hearing loss unilateral, Inner ear infection, Muffled hearing in both ears.
5. **Environmental Exposure**: Accidental exposure to product, Accidental exposure to product by child, Allergic reaction, Allergy, Anaphylactic type reaction, Latex allergy, Lead poisoning, Nicotine poisoning, Pesticide poisoning, Poisoning, Toxic reaction (NOS), Whipworm infection.
6. **Eyes**: Dilated pupils, Dry eyes, Eye abnormality, Eye irritation, Eye rolling, Eye strain, Eyes rolling, Irritation of eyes, Low vision-one eye, Nearsighted, Retinal drusen, Spots before eyes, Vision abnormal, Vision decreased.
7. **Legal/Social Issues**: Apparent life-threatening event, Disability NOS, Driving ability disturbed, Impaired work ability, Inability to work, Legal problem, Near death experience.
8. **Metabolic/Endocrine System**: Diabetes, Lactic acid, Secondary hypothyroidism.
9. **Reproductive System**: Adenocarcinoma of the prostate recurrent, Endometriosis, Menopausal, Obstetrical pulmonary embolism, Vaginal discharge, Vaginal yeast infection, Vulval itching, Vulvitis.
10. **Substance Use**: Cigar smoker, Cigarette smoker, Drug withdrawal syndrome in newborn, E-cigarette smoker.

Appendix 3. Cohen's weighted kappa for each health category.

Category	Cohen's Weighted Kappa [95% CI]	Std. Error	*K* Level of Agreement
Respiratory	0.977 [0.933, 1.022]	0.023	Near perfect agreement
Psychological	0.845 [0.634, 1.056]	0.108	Near perfect agreement
Neurological	0.976 [0.930, 1.023]	0.024	Near perfect agreement
Cardiovascular	1.000 [1.000, 1.000]	0.000	Near perfect agreement
Oral/Dental	1.000 [1.000, 1.000]	0.000	Near perfect agreement
Integumentary	0.794 [0.402, 1.187]	0.200	Substantial agreement
Digestive	0.950 [0.853, 1.047]	0.050	Near perfect agreement
Musculoskeletal	1.000 [1.000, 1.000]	0.000	Near perfect agreement
Physical Trauma	0.851 [0.565, 1.138]	0.146	Near perfect agreement
Nonspecific	0.945 [0.871, 1.020]	0.038	Near perfect agreement

Appendix 4. Adverse health events by year.

Year	Frequency	Percent
2017	47	5.6
2018	60	7.2
2019	551	65.7
2020	98	11.7
2021	62	7.4
2022	21	2.5
**Total**	**839**	**100.0**
